# problematic social media use among child and adolescent psychiatry consultants: family risk factors

**DOI:** 10.1192/j.eurpsy.2022.1104

**Published:** 2022-09-01

**Authors:** A. Ben Othman, M. Hamza, B. Amemou, A. Ben Hamouda, S. Bourgou, F. Charfi, A. Belhadj

**Affiliations:** 1 Mongi Slim University Hospital, Child And Adolescent Psychiatry Department, La Marsa, Tunisia; 2 Fattouma Bourguiba University Hospital, Psychiatry Department, Monastir, Tunisia; 3 hospital of mongi slim, Pedopsychiatry, Tunis, Tunisia

**Keywords:** APGAR Family Test, social media, Child and adolescent, Bergen Social Media Addiction Scale

## Abstract

**Introduction:**

the problematic use of social media (PUSM) is considered nowadays as a behavioural addiction. Social media seem to provide an ephemeral escape especially for children suffering from dysfunctional families and abuse.

**Objectives:**

To study in a population of children and adolescents followed in outpatient child psychiatry unit, the prevalence, and family risk factors related to PUSM.

**Methods:**

a descriptive study was conducted among child and adolescent psychiatry consultants. Parents were asked to provide answers for the BSMAS (Bergen Social Media Addiction Scale). We used a self-administered questionnaire and the BSMAS to assess patients’ social media’s use characteristics and the APGAR Family Test to assess their satisfaction with their family functioning.

**Results:**

The prevalence of PUSM was estimated at 9.2% in our population according to the conservative approach, rising to 48.7% according to the liberal approach. APGAR Family Test scores were negatively correlated with BSMAS scores (Pearson’s coefficient= -0.37; p=0.002). Significantly higher scores were found in cases of exposure to physical (p=0.001) or moral (p=0.037) abuse and among patients who witnessed spousal violence (p=0.041), and whose parents had a lower level of education. A positive and significant correlation was found between parents’ and adolescents’ BSMAS scores (p=0.04).

**Conclusions:**

Psychopathological fragility triggered by poor family functioning expose to the risk of PUSM. The implementation of preventive strategies and a rigorous and global management of these adolescents are imperative to fight against this disorder.

**Disclosure:**

No significant relationships.

